# Ball-Valve Syndrome Secondary to Large Fundic Adenoma

**DOI:** 10.14309/crj.0000000000001330

**Published:** 2024-04-10

**Authors:** Hyun Jae Kim, Andrew Fetz, David Sanders, Emile Woo, Eric Lam

**Affiliations:** 1 Department of Gastroenterology, University of British Columbia, Vancouver, British Columbia, Canada; 2 Department of Surgery, University of British Columbia, Vancouver, British Columbia, Canada

**Keywords:** Intussusception, prolapse, ball valve, fundic adenoma, gastric mass

## Abstract

Gastroduodenal intussusception is a rare presentation in adults. A mass lesion in the stomach typically acts as a lead point that invaginates into the pylorus and duodenum causing intussusception. In a subset of these cases, episodic symptoms of obstruction occur because of intermittent prolapse of the lesion, termed “ball-valve syndrome.” We present a 73-year-old woman with intermittent abdominal pain and nausea who was discovered to have gastroduodenal intussusception secondary to a large prolapsing fundic adenoma through the pylorus and into the duodenum. The case highlights this rare complication from gastric lesions along with the importance of surgical intervention for definitive management.

## INTRODUCTION

Gastroduodenal intussusception in adults is a very rare occurrence characterized by telescoping of the gastric fundus or body into the duodenum. Typically, a tumorous lesion in the stomach acts as a lead point that invaginates into the pylorus and duodenum. We describe a case of a large fundic adenoma leading to gastroduodenal intussusception, also known as the ball-valve syndrome, and discuss the importance of surgical intervention in management of this rare condition.

## CASE REPORT

A 73-year-old woman was referred to a community gastroenterology office for 6 weeks of intermittent abdominal pain and nausea, associated with a weight loss of 10 pounds. There was no reported history of overt gastrointestinal bleeding. She had a history of cutaneous basal cell carcinoma that was resected remotely but was otherwise healthy and denied any history of smoking or excessive alcohol intake. Her bloodwork was significant for iron deficiency anemia with hemoglobin of 78 g/L and ferritin of 6 μ/L. Other cell counts, electrolytes, thyroid-stimulating hormone, vitamin B12, and renal function were normal.

**Figure 1.**
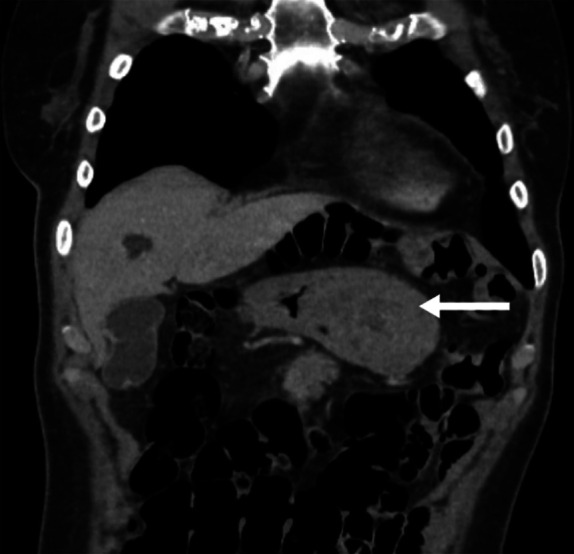
Computed tomography of the abdomen/pelvis demonstrated 6-cm fundic mass invaginating into the antrum (arrow).

Esophagogastroduodenoscopy was subsequently performed, which demonstrated a large 7-cm pedunculated lesion in the gastric fundus. The large gastric lesion was prolapsing through the pylorus into the duodenum but was able to be brought back into the stomach using biopsy forceps. Biopsies of the lesion were consistent with gastric tubular adenoma with low-grade dysplasia. Notably, the patient had temporary symptom relief after endoscopic reduction. Computed tomography scan confirmed a 6-cm gastric fundic mass prolapsing into the antrum (Figure 1). There was no evidence of metastatic disease. On radial endoscopic ultrasound, the lesion was mucosal-based without submucosal invasion. Because of the size of the lesion, she was referred to a tertiary center for endoscopic submucosal dissection (ESD). ESD was attempted; however, the location of the polyp stalk at fornix of the fundus as well as distorted anatomy made it technically challenging. Hence, ESD was aborted, and she was referred for surgical resection.

A single-port laparoscopy-assisted intragastric polypectomy was considered as a preferred approach. She was planned for elective surgery 2 months later. However, during this time, she began to deteriorate with increasing difficulty with oral intake because of intermittent obstructions. One week before her planned surgical date, she was admitted to her local hospital because of hypovolemia with inability to tolerate oral intake. She was resuscitated and started on total parenteral nutrition and was subsequently transferred to our center for surgery.

**Figure 2.**
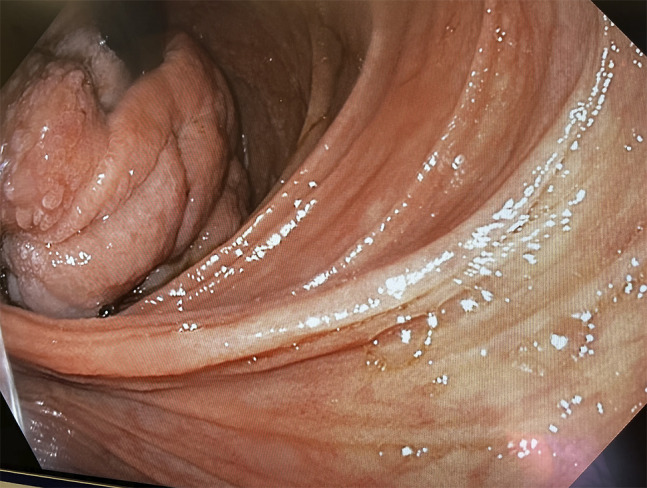
Retroflexed endoscopic view of the prolapsing fundic mass in the duodenum.

**Figure 3.**
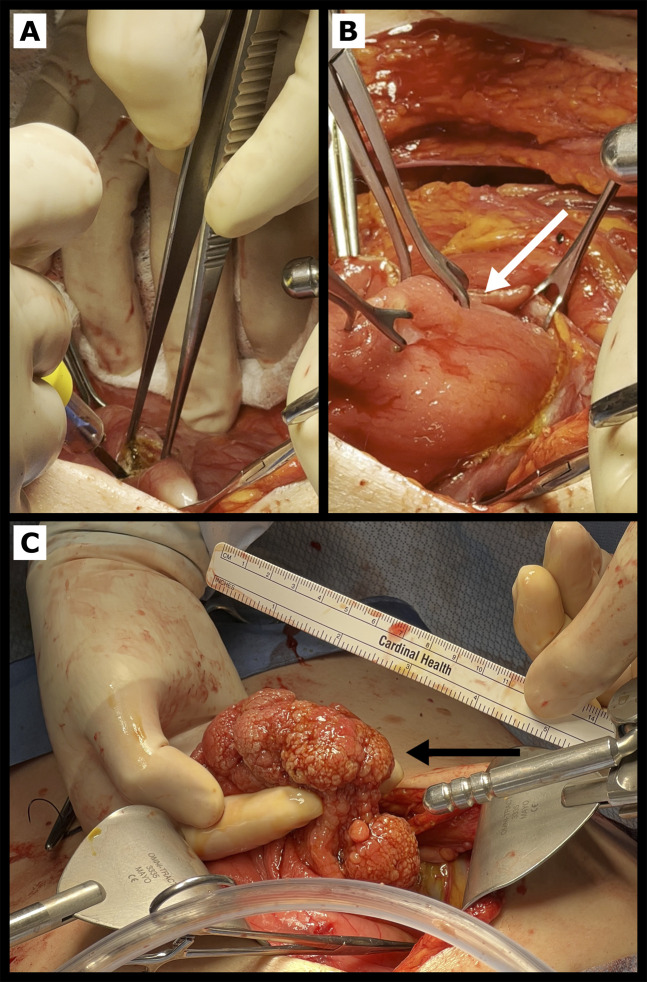
Intraoperative findings during open laparotomy for gastroduodenal intussusception. (A) Distal gastrotomy is performed to assist in reducing gastroduodenal intussusception. (B) Using grasper forceps, gentle continuous pulling pressure is applied to the intussuscepting gastric body (white arrow) to reduce gastric lead point into the stomach. (C) After reduction, large 30 × 80-mm gastric adenoma (black arrow) in gastric fundus was confirmed to be acting as a lead point.

Intraoperative esophagogastroduodenoscopy was performed, which demonstrated gastroduodenal intussusception with polyp prolapsing into the duodenum (Figure 2). It was not endoscopically reducible. Single-port intragastric surgery and subsequent laparoscopic resection were attempted; however, the surgery was converted to open laparotomy because of distorted anatomy from intussusception and associated inflammation. Large gastroduodenal intussusception with proximal gastric polyp acting as a lead point was confirmed. The upper one-third of the stomach along the greater curvature was visualized prolapsing into the duodenum. Distal gastrotomy was performed, and polyp resection was performed using linear stapler through the gastrotomy (Figure 3). The resected polyp measured 8 × 3 cm. Once the polyp was resected, the intussusception spontaneously reduced and the gastrotomy site was closed.

Her postoperative course was complicated by delayed return of her gastric function and minor cutaneous midline wound separation, but otherwise, she recovered well and was discharged on postoperative day 14. Histopathology examination of resected tissue confirmed gastric adenoma with low-grade and focal high-grade dysplasia. On follow-up, the patient has been doing well without any recurrence of symptoms.

## DISCUSSION

Gastrointestinal intussusception in adults is rarely seen with an incidence of 2–3 cases per million adults per year and accounts for less than 1% of gastrointestinal obstructions.^1^ Intussusceptions can occur in any part of the gastrointestinal tract, but majority (90%) occur in the small or large bowel. Gastroduodenal intussusception is an extremely rare event and accounts for less than 10% of all intussusceptions.^2^

**Figure 4.**
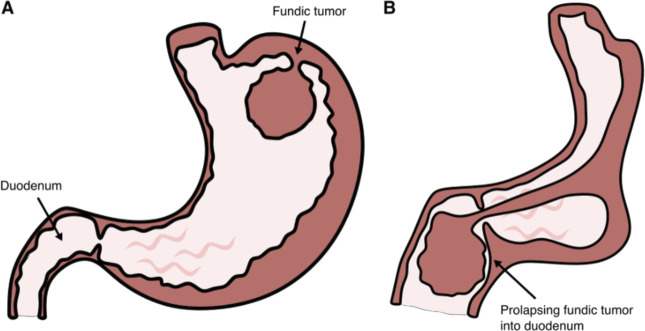
Graphical illustration demonstrating mechanism of gastroduodenal intussusception secondary to fundic mass. (A) Fundic polyp acts as a lead point and (B) initiate the invagination process causing gastroduodenal intussusception.

Unlike in pediatric population, intussusceptions in adult patients are rarely idiopathic, and an identifiable cause can usually be found. Typically in gastroduodenal intussusceptions, a polypoidal mass acts as a lead point and prolapse into the pylorus and duodenum as seen in our case (Figure 4).^3,4^ Mucosal polyps and adenocarcinoma along with submucosal tumors, such as gastrointestinal stromal tumor and lipoma, are common culprits.^5^ Based on review of 143 cases of gastroduodenal intussusceptions caused by gastric tumors in Japan, most tumors were in antrum (68%) and gastric body (25%), whereas gastroduodenal intussusceptions from a fundic tumor, as was our case, were rare (7%).^6^

Presentations of gastroduodenal intussusception vary based on the degree of obstruction. Commonly reported symptoms include epigastric pain, vomiting, anemia, gastrointestinal bleeding, and abdominal distention. In severe and delayed presentations, bowel ischemia and peritonitis have been reported.^2,4,7^ Not uncommonly patients can present with periodic and intermittent symptoms caused by intermittent prolapse of gastric lesion through the pylorus. This is described as the ball-valve syndrome. This was the case with our patient with evidence of a prolapsing adenoma, “ball,” in and out of the pylorus, “valve,” at different times. The intermittent and nonspecific nature of symptoms makes the diagnosis elusive. Preoperative diagnosis of gastroduodenal intussusception typically involves cross-sectional imaging and endoscopy.^8^

Treatment of gastroduodenal intussusception is primarily surgical. Although endoscopic reduction of intussusception can be performed, definitive treatment of gastroduodenal intussusception requires surgical resection of the causative gastric lesion. Few cases of successful endoscopic resection through ESD have been reported, but surgical resection is preferred because of (i) significant distortion of anatomy making ESD challenging, (ii) risk of malignant etiology, and (iii) possibility of bowel ischemia that can be assessed at the time of surgery.^9^

In summary, we present a case of a large fundic adenoma causing gastroduodenal intussusception, ball-valve syndrome, which was treated with surgical reduction and excision after failed endoscopic resection attempt. Endoscopic reduction and resection can be considered, but gastroduodenal intussusception usually requires surgical intervention because of distorted anatomy and malignancy risk.

## DISCLOSURES

Author contributions: HJ Kim: manuscript write-up and is the article guarantor; A. Fetz, D. Sanders, E. Woo, and E. Lam: edited manuscript provided endoscopic/surgical images.

Financial disclosure: None to report.

Informed consent was obtained for this case report.
